# Proteome of granulosa cells lipid droplets reveals mechanisms regulating lipid metabolism at hierarchical and pre-hierarchical follicle in goose

**DOI:** 10.3389/fvets.2025.1544718

**Published:** 2025-03-31

**Authors:** Xin Yuan, Xi Zhang, Yueyue Lin, Hengli Xie, Zhujun Wang, Xinyue Hu, Shenqiang Hu, Liang Li, Hehe Liu, Hua He, Chunchun Han, Xiang Gan, Ling Liao, Lu Xia, Jiwei Hu, Jiwen Wang

**Affiliations:** ^1^College of Animal Science and Technology, Sichuan Agricultural University, Chengdu, China; ^2^Scientific Research Center, Guilin Medical University, Guilin, Guangxi, China; ^3^College of Horticulture, Sichuan Agricultural University, Chengdu, China

**Keywords:** goose, granulosa cell, lipid metabolism, lipid droplet, lipid droplet-associated proteins

## Abstract

Avian hierarchical follicles are formed by selection and dominance of pre-hierarchical follicles, and lipid metabolism plays a pivotal role in this process. The amount of lipid in goose follicular granulosa cells increases with the increase of culture time, and the neutral lipid in the cells is stored in the form of lipid droplets (LDs). LD-associated proteins (LDAPs) collaborate with LDs to regulate intracellular lipid homeostasis, which subsequently influences avian follicle development. The mechanism by which LDAPs regulate lipid metabolism in goose granulosa cells at different developmental stages is unclear. Therefore, using BODIPY staining, we found that at five time points during *in vitro* culture, the LD content in hierarchical granulosa cells was significantly higher than that in pre-hierarchical granulosa cells in this study (*p* < 0.001). Next, we identified LDAPs in both hierarchical and pre-hierarchical granulosa cells, and screened out 1,180, 922, 907, 663, and 1,313 differentially expressed proteins (DEPs) at the respective time points. Subsequently, by performing Clusters of Orthologous Groups (COGs) classification on the DEPs, we identified a large number of proteins related to lipid transport and metabolism. Following this, the potential functions of these DEPs were investigated through Gene Ontology (GO) and Kyoto Encyclopedia of Genes and Genomes (KEGG) functional enrichment analysis. Finally, the important pathway of fatty acid degradation and the key protein ACSL3 were screened out using Short Time-series Expression Miner (STEM) and Protein–Protein Interaction (PPI) analysis methods. It is hypothesized that ACSL3 may potentially modulate lipid metabolism through the fatty acid degradation pathway, thereby contributing to the difference in lipid content between hierarchical and pre-hierarchical granulosa cells. These findings will provide a theoretical foundation for further studies on the role of LDs and LDAPs in avian follicle development.

## 1 Introduction

Lipids not only provide energy for follicle development but also serve as important regulatory factors in this process (1). A mature follicle usually consists of oocyte, granulosa, theca and connective tissue layer (2). The granulosa cell is the main component of the follicular wall and is one of the most important structures of the follicle (3, 4). Recently, lipid metabolism in granulosa cells has attracted attention. Studies on mice have shown that treating granulosa cells with palmitic acid (PA) or lysophosphatidylcholine (LPC) reduces cell viability and induces apoptosis (5, 6). Oleic acid (OA) alters lipid storage in porcine ovarian granulosa cells and reduces steroidogenesis (7). Furthermore, arachidonic acid (AA), a polyunsaturated fatty acid, has a significant impact on granulosa cell function and follicle development. Research has found that low doses of AA can induce the accumulation of low-density lipoprotein in bovine granulosa cells and enhance granulosa cell survival (8). Granulosa cells also play a pivotal role in follicle development and maturation. During follicle growth, maturation, and ovulation, granulosa cells transport nutrients to the follicle, provide mechanical support, and synthesize steroids (9, 10). Undeniably, lipid metabolism in granulosa cells is crucial for the development of mammalian follicles.

Unlike mammalian follicular development, avian follicular development exhibits a unique hierarchical system, including hierarchical follicles and pre-hierarchical follicles. During follicular development, only a very small number of pre-hierarchical follicles can develop into hierarchical follicles (11). There are significant differences in tissue morphology and steroid secretion between hierarchical and pre-hierarchical follicles (12, 13). Recent studies have found that there are also differences in lipid metabolism between hierarchical and pre-hierarchical granulosa cells, with the ability of hierarchical granulosa cells to accumulate lipids being stronger (14). Research using mass spectrometry on the lipid profile of granulosa cells also suggests that granulosa cell lipids are important for follicular development (15). Neutral lipids in granulosa cells are stored in the form of lipid droplets (LDs). LDs, ubiquitous lipid-storing organelles (16, 17), participate in diverse cellular processes like energy metabolism (18), lipid storage, transport, and metabolism (19, 20), as well as protein storage and degradation (21, 22). The functionality of LDs is heavily reliant on the associated proteins, which are collectively referred to as lipid droplet-associated proteins (LDAPs). LDs have been isolated from human (23), mouse (24, 25), pig, fish (26, 27), and hundreds of proteins associated with LDs have been reliably detected by means of proteomic (28). LDAPs are involved in the regulation of cellular lipid metabolism in concert with LDs. It has been found that LDs contain a large number of LDAPs related to lipid metabolism, such as Perilipins (PLIN) (29), Acyl-CoA synthetase proteins (ACSL) (30), Adipose triglyceride lipase (ATGL) (31), and these proteins further regulate the process of lipid metabolism through the way of promoting the synthesis and degradation of LDs.

Lipid metabolism in granulosa cells is crucial for follicular development (13), yet the regulation of LDAPs in avian granulosa cells remains understudied, and there is currently no report on the proteome of LDs in geese. Therefore, this study first examined the differences in LD content in granulosa cells at various developmental stages through LD staining. Subsequently, LDs were isolated, and their associated proteins were identified using proteomics. Lastly, proteomic analysis was employed to delve into the molecular mechanisms underlying the regulation of lipid metabolism in granulosa cells by LDAPs.

## 2 Materials and methods

### 2.1 Animals

Healthy female lines of Tianfu geese (*Anser cygnoides*) were selected. Food and water were provided to the geese under natural light and temperature conditions at the Waterfowl Breeding Experimental Farm of Sichuan Agricultural University (Ya’an, China). The spawning cycle of each goose was recorded, and ovarian follicles were collected from all geese during the same spawning cycle. All selected geese were euthanized by carbon dioxide inhalation and cervical dislocation performed by competent, experienced personnel who applied the techniques correctly. Efforts were made to minimize animal suffering in accordance with the requirements of the Beijing Animal Welfare Committee. All experimental procedures involving animal manipulation were approved by the Committee of the School of Farm Animal Genetic Resources Exploration and Innovation Key Laboratory, College of Animal Science and Technology, Sichuan Agricultural University, under permit no. DKY20170913, and were conducted in accordance with the Regulations of the Administration of Affairs Concerning Experimental Animals (China 1988).

### 2.2 Cells culture and incubation conditions

For granulosa cell culture, after euthanasia of 18 randomly selected geese with regular egg production, follicles were collected from the coelomic cavity of goose, placed in sterile saline, and divided into hierarchical (F2-F4 large yellow follicles) and pre-hierarchical follicles (small follicles between 8 and 10 mm in diameter) according to previously reported nomenclature (32). The outer connective tissue was removed from the follicles, and follicles were bisected to allow the yolk and adherent granulosa layer to flow out (33). Collecting the granulosa layer, incubated with 0.05% type II collagenase (Sigma, USA) for 10 min in a 37°C water bath, dispersed, resuspended in 3 mL of fresh collagenase-free basal medium, and centrifuged. Cells were then dispersed in Dulbecco’s modified Eagle medium (DMEM) supplemented with 1% antibiotic/antimycotic solution (Solarbio, Beijing, China) and 5% fetal bovine serum (Gibco, Waltham, MA, USA). Then, cells were incubated in a water-saturated atmosphere of 95% air and 5% CO2 at 37°C in an incubator (Thermo, Waltham, MA, USA), as previously described (34).

### 2.3 BODIPY staining of lipids in granulosa cells

The cell samples were rinsed 2–3 times with phosphate-buffered saline (PBS) to remove the medium. 4% paraformaldehyde solution was added to cover the cell samples, and fixation was carried out for 30 min at room temperature. After fixation, the cell samples were rinsed 2–3 times with PBS. The BODIPY staining solution was then added, and the samples were incubated for 15 min. The cell samples were washed 2–3 times with PBS to remove unbound staining solution. The DAPI staining solution was added, and the samples were incubated for 15 min at room temperature, followed by rinsing 2–3 times with PBS. Finally, the cell samples were observed under a fluorescence microscope using appropriate fluorescent filters. The images were further analyzed and processed using the ImageJ software package. The area of the LDs (green) and the area of the nucleus (blue) were measured using the software, and the LDs area/nucleus area was calculated as the relative fluorescence ratio. Three biological replicates, each with three technical replicates (*n* = 9).

### 2.4 LDs isolation from granulosa cells

LDs were isolated from granulosa cells according to the manufacturer’s instructions (Cell Biolabs, INC. cat#MET-5011). Briefly, 1.5 × 10^7^ granulosa cells were digested, and the medium was removed by first washing the cells with PBS. After washing, the cells were resuspended in 200 μL of Reagent A, incubated on ice for 10 min, and then 800 μL of Reagent B was added. The cells were homogenized by passing them through a 27-gauge needle five times. 600 μL of Reagent B are cautiously added to the homogenate and centrifuged at 18,000 g at 4°C for 3 h. Subsequently, the uppermost 270 μL of the stratified solution were aspirated to yield the LDs.

### 2.5 DEPs sample preparation for LC-MS/MS analysis

LDs were isolated from hierarchical and pre-hierarchical granulosa cells after 2 d, 3 d, 4 d, 5 d, and 6 d of culture, respectively. Three technical replicates were performed for each time point (*n* = 15). LDs isolated from hierarchical granulosa cells after 2 d of culture were referred to as the H2 group, while LDs from pre-hierarchical granulosa cells after 2 d of culture were referred to as the P2 group. The remaining samples were named sequentially. Total protein amount for each sample was at least 30 μg, proteomic analysis of LD proteins from each sample was performed by mixing a specific concentration of formic acid with the digested sample, adjusting the pH below 3, and centrifuging at 12,000 g for 5 min at room temperature. The samples were then desalted using a C18 desalting column, and the eluate was collected and lyophilized.

The spectra library for data-independent acquisition (DIA) was constructed using UHPLC–MS/MS in Novogene Bioinformatics Technology Co., Ltd. (Beijing, China). To build the library, LD proteomics analysis was performed using a Vanquish Neo upgraded UHPLC system (Thermo Fisher, America), and the separated peptides were analyzed using a Thermo Orbitrap Fusion Lumos mass spectrometer (Thermo Fisher, America). To improve the quality of the analytical results, the DIA-NN software further filtered the search results by retaining only credible Peptide Spectrum Matches (PSMs) with a confidence level of 99% or higher.

### 2.6 Protein identification and quantification

The raw files were searched and analyzed using the DIA-NN library search software, based on the UniProt database specific to the *Anser cygnoides* proteome. Only credible spectral peptides and proteins were retained, and FDR validation was performed to remove peptides and proteins with an FDR greater than 1%. Proteins were quantified in all three replicate samples. Proteins with a peptide number greater than 2 were selected for further analysis. Significantly differentially expressed proteins (DEPs) were screened according to the criteria with fold change >1.5 (up-regulation) or < 0.67 (down-regulation) and *p* < 0.05.

### 2.7 Bioinformatic analysis

The original functions of the proteins were clustered using Clusters of Orthologous Groups (COGs) analysis.^1^ Gene Ontology (GO) functional annotation was performed using InterProScan software, with reference to the chicken (*Gallus gallus*) database. The functions of different proteins were categorized using GO assignment^2^ to classify the identified proteins into three main categories: biological processes, cellular components, and molecular functions. The most important biochemical metabolic pathways and signal transduction pathways of the proteins were identified using the Kyoto Encyclopedia of Genes and Genomes (KEGG) pathway.^3^ Short Time-Series Expression Miner (STEM) clustering algorithm was used to identify temporal expression profiles of LD proteins. Representative temporal expression profiles were defined as model profiles, and protein expression values were transformed into logarithmic ratios of expression relative to Day 2. Each protein was then assigned to a model profile based on the screening criteria, and the correlation coefficients between the protein’s expression profile and the model profile were determined. If the correlation coefficients indicate statistical significance, the corresponding data points or boxes in the plot are colored. Interactions between proteins were analyzed by constructing PPI networks. The interaction information of all proteins was collected using the online database STRING,^4^ and the results were imported into Cytoscape for visualization.

### 2.8 Statistical analysis

For each experimental procedure, data were collected simultaneously from at least three geese per group, with at least three technical repetitions set up. The data were expressed as mean ± standard error of the mean (SEM), significant differences between the proteins in the two phases of LDs were determined using independent t-tests and one-way ANOVA (GraphPad Prism 5), the obtained data were initially analyzed for normal distribution using descriptive statistics in SPSS, followed by significance analysis using t-tests and one-way ANOVA (SPSS version 20.0, IBM, Armonk, IL, USA). Differences with *p* < 0.05 were considered statistically significant, images drawn using Prism 5, Graphpad software. Metabolic pathway plots were drawn using Adobe Photoshop.

## 3 Results

### 3.1 Differences of LDs content *in vitro* cultured granulosa cells from geese follicles at different developmental stages

**Figure 1:**
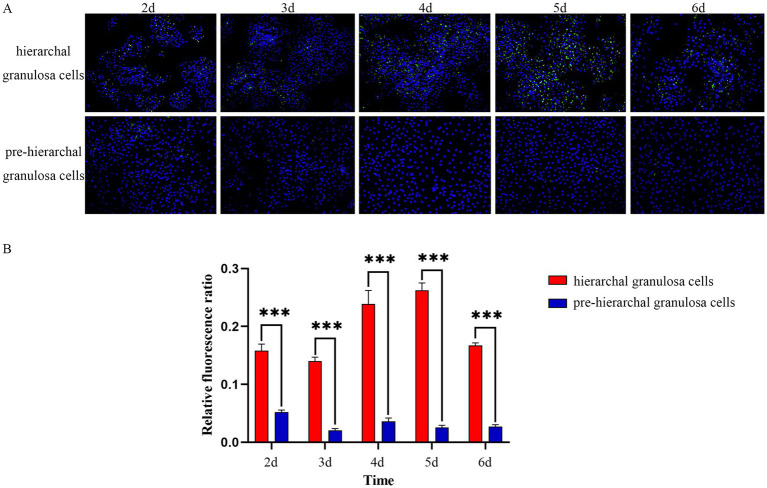
BODIPY staining of LDs. **(A)** Morphological characteristics of LDs in granulosa cells detection by BODIPY (green) and DAPI (blue) staining. **(B)** With increasing incubation time, the LDs content in hierarchical granulosa cells and pre-hierarchical granulosa cells showed dynamic changes, and the LDs content in hierarchical granulosa cells was significantly higher than that in pre-hierarchical granulosa cells. ***p < 0.001.

Using BODIPY staining, we investigated the LD content in two distinct groups of granulosa cells, isolated from follicles at different developmental stages. As shown in Figure 1, the results indicated that LDs of granulosa cells were spherical in shape, and with increasing incubation time, the hierarchical granulosa cells tended to accumulate larger LDs, in contrast to the smaller droplets formed by pre-hierarchical granulosa cells. The staining results indicate significant differences in LD content between granulosa cells at different developmental stages. Specifically, the LD content in hierarchical granulosa cells is significantly higher than that in pre-hierarchical granulosa cells (*p* < 0.001). To gain further insights into the underlying mechanisms, we subsequently purified high-quality LDs from granulosa cells for comprehensive proteomic analysis.

### 3.2 Identification and classification of LDAPs

**Figure 2:**
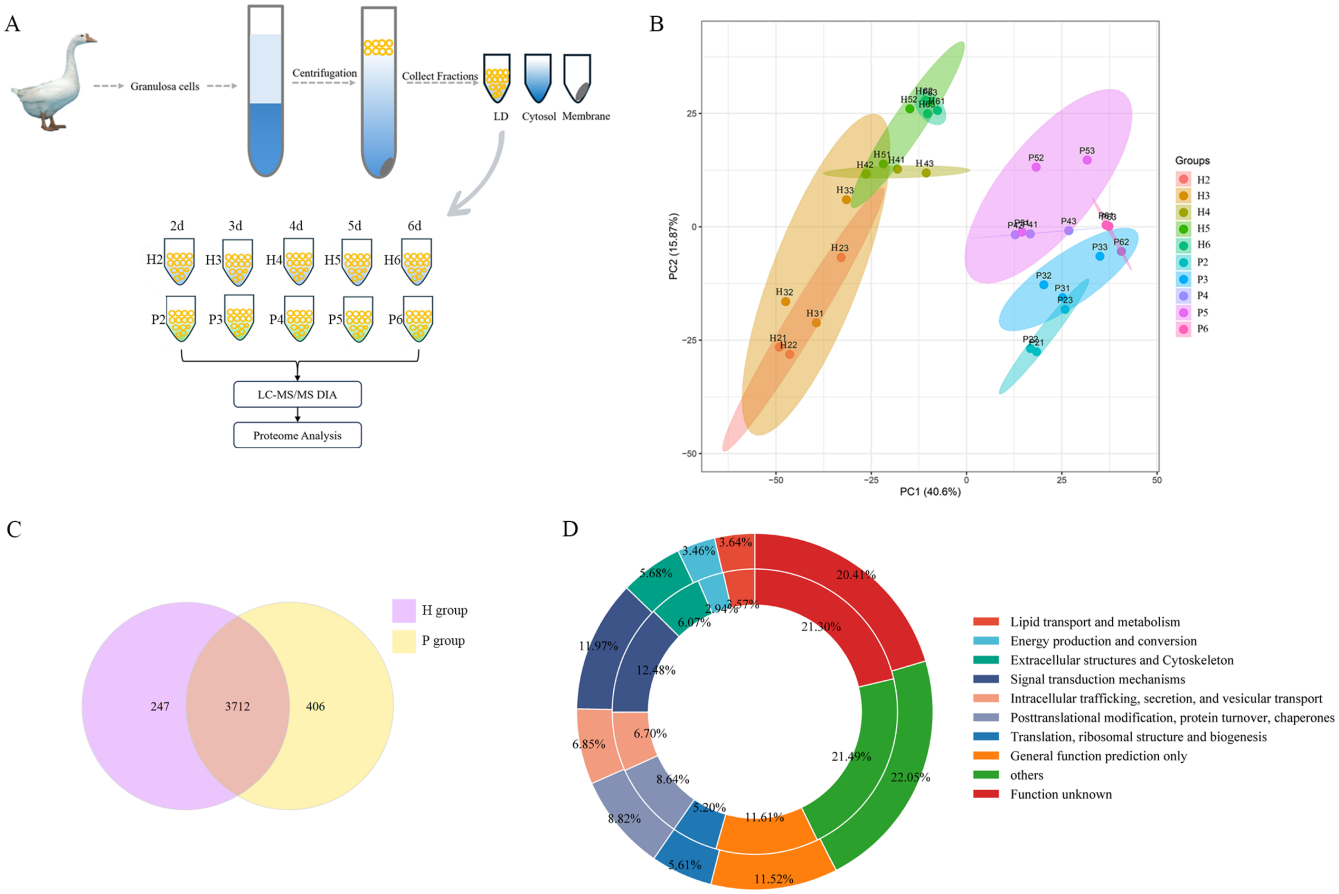
Identification and classification of LDAPs. **(A)** Schematic of protein sample collection. **(B)** PCA analysis of all proteins. **(C)** The Venn diagram showed overlapping proteins between the H and P groups proteomes.**(D)** The 4,365 proteins of LDs identified in two groups samples were categorized into 10 groups: Lipid transport and metabolism; Energy production and conversion; Extracellular structures and Cytoskeleton; Signal transduction mechanisms; Intracellular trafficking, secretion, and vesicular transport; Posttranslational modification, protein turnover, chaperones; Translation, ribosomal structure and biogenesis; General function prediction only; others and Function unknown protein. Circles from outer to inner represent H and P groups.

Proteomic studies were conducted to gain insights into the LDAPs in granulosa cells from hierarchical and pre-hierarchical follicles, a schematic workflow is shown in Figure 2A. The application of Principal Component Analysis (PCA) to the amassed proteomic dataset reveals distinct separation between hierarchical granulosa cells hierarchical granulosa cells (H group) and pre- hierarchical granulosa cells (P group), as shown in Figure 2B. Comparison with the UniProt database identified and quantified a total of 4,365 LDAPs. Of these, 3,959 LDAPs were identified from the H group and 4,118 from the P group, with 3,712 proteins co-identified in both groups (Figure 2C). To further analyze these proteins, we performed a Cluster of Orthologous Groups (COGs) analysis, which categorized the 4,365 proteins into 10 groups based on their cellular functions using the COGs database. As shown in Figure 2D and Supplementary Table S1, we found that many proteins were enriched in the COGs category ‘lipid transport and metabolism’, including numerous enzymes related to lipid synthesis and degradation, such as Fatty acid synthase (FASN), Fatty acid-binding proteins (FABP1, FABP4, FABP7), long-chain acyl-coenzyme A synthases (ACSL1, ACSL3, ACSL4), Acyl-CoA synthetase family members (ACSF2, ACSF3), Methylsterol monooxygenase 1 (MSMO1), Diacylglycerol kinases (DGKD, DGKQ), and Monoglyceride lipase (MGLL).

### 3.3 DEPs between hierarchical and pre-hierarchical granulosa cells

**Figure 3:**
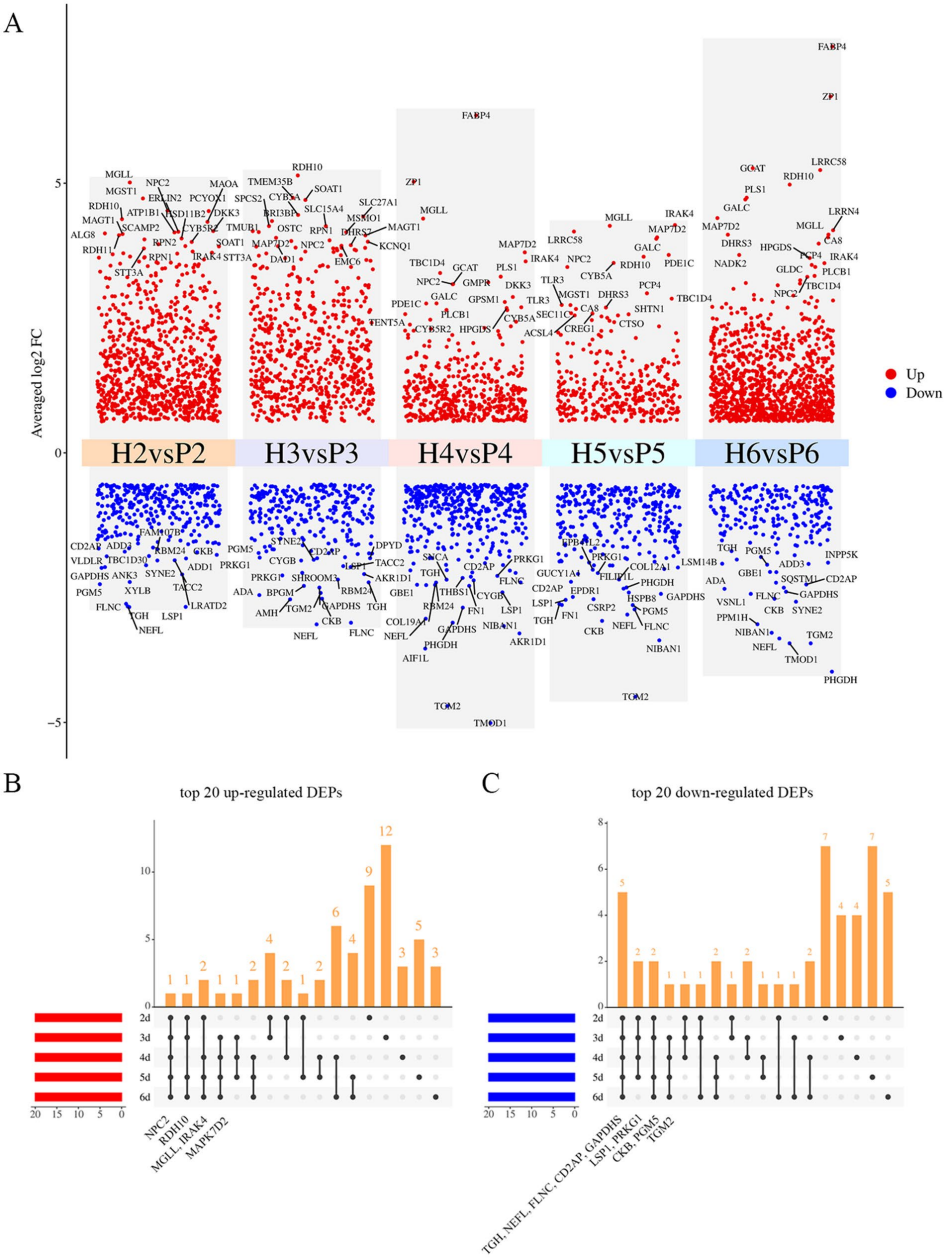
Significantly changed proteins at five times points. **(A)** Volcano plot of DEPs at five time points, top 20 up- and down-regulated DEPs are labeled in the figure, red and blue show up- and down-regulated proteins. **(B)** Upset plot of top 20 up-regulated DEPs at five time points, the annotated proteins were those significantly up-regulated and ranked within the top 20 at all 4 or 5 time points. **(C)** Upset plot of top 20 down-regulated DEPs at five time points, the annotated proteins were those significantly down-regulated and ranked within the top 20 at all 4 or 5 time points.

We further analyzed the DEPs in the H and P groups across five time points. At 2 d of cell culture (H2 vs. P2), a total of 1,180 DEPs were identified, with 856 DEPs up-regulated and 324 DEPs down-regulated. At 3 d (H3 vs. P3), 922 DEPs were identified, comprising 640 up-regulated and 282 down-regulated proteins. Similarly, at 4 d (H4 vs. P4), 907 DEPs were identified, including 468 up-regulated and 439 down-regulated. At 5 d (H5 vs. P5), 663 DEPs were screened, with 356 up-regulated and 307 down-regulated. Lastly, at 6 d (H6 vs. P6), 1,313 DEPs were screened, of which 1,072 were up-regulated and 241 were down-regulated (Figure 3A). Notably, NPC intracellular cholesterol transporter 2 (NPC2) was consistently among the top 20 up-regulated DEPs across all five time points. Monoglyceride lipase (MGLL), Retinol dehydrogenase 10 (RDH10), and Interleukin-1 receptor-associated kinase 4 (IRAK4) were also among the top 20 up-regulated DEPs in four of the time points. Conversely, five DEPs, including Carboxylic ester hydrolase (TGH), Neurofilament light (NEFL), Filamin C (FLNC), Peptidyl-cysteine S-nitrosylase GAPDH (GAPDHS), and CD2 associated protein (CD2AP) were consistently among the top 20 down-regulated DEPs across all five time points. Lymphocyte-specific protein 1 (LSP1), Phosphoglucomutase 5 (PGM5), cGMP-dependent protein kinase (PRKG1), Creatine kinase B (CKB), and Transglutaminase 2 (TGM2) were also among the top 20 down-regulated DEPs in four of these time points (Figure 3B; Supplementary Table S2).

### 3.4 Pathway enrichment analysis of DEPs

To elucidate the potential functions of these proteins, we performed GO and KEGG pathway enrichment analysis (Supplementary Table S3). In the five time points, BP was enriched for a large number of biological processes related to fatty acid metabolism, such as fatty acid metabolism, fatty acid biosynthesis, and fatty acid *β*-oxidation. CC was enriched for cellular components such as LDs, mitochondria, endoplasmic reticulum, and MF was enriched for molecular functions such as lipid-binding, calcium-dependent phospholipid-binding, and phospholipid-binding. The five groups of KEGG pathways were enriched in 114 metabolic pathways, of which 23 were all related to lipid metabolism (Table 1). Five of these pathways were significant in each group, namely fatty acid metabolism, fatty acid degradation, fatty acid biosynthesis, glycerophospholipid metabolism, and PPAR signaling pathway. The top 20 most significantly enriched GO terms and KEGG pathways in the five time points, according to the smallest to largest *P*, are shown in Supplementary Figure S1.

**Table 1:** 23 pathways related to lipid transport and metabolism in KEGG.

KEGG pathway	H2vsP2		H3vsP3		H4vsP4		H5vsP5		H6vsP6	
	*P*	Protein number	*P*	Protein number	*P*	Protein number	*P*	Protein number	*P*	Protein number
Adipocytokine signaling pathway	0.081903718	6	0.399736772	3	0.894016652	1	0.496527864	2	0.238992617	5
Arachidonic acid metabolism	0.391842125	3	0.259843213	3	0.104576542	4	0.720128934	1	0.11790552	5
Biosynthesis of unsaturated fatty acids	0.004129396	6	0.006264012	5	0.100075282	3	0.545797932	1	0.079336257	4
Ether lipid metabolism	0.059640273	5	0.024985137	5	0.212907204	3	0.322021909	2	0.086035527	5
Fatty acid metabolism	8.61185E-10	18	6.88941E-09	15	4.37861E-08	14	0.000743567	7	1.47597E-10	20
Fatty acid degradation	4.63435E-08	13	1.36009E-06	10	1.31832E-06	10	0.000395103	6	7.99851E-11	17
Fatty acid elongation	0.001875318	6	0.016049957	4	0.015866054	4	0.484105001	1	0.000693086	7
Fatty acid biosynthesis	0.011553426	4	0.000731424	5	0.004949534	4	0.013081478	3	0.00061686	6
FoxO signaling pathway	0.063064124	10	0.929063184	2	0.01525887	10	0.219977859	5	0.187334174	9
Glycerophospholipid metabolism	7.08801E-05	15	7.06706E-05	13	0.022661473	8	0.004206846	8	0.028526744	10
Glycerolipid metabolism	0.008863215	8	0.173087583	4	0.02420311	6	0.006225667	6	0.042033529	7
Inositol phosphate metabolism	0.051616502	7	0.251519932	4	0.005276411	8	0.003455361	7	0.000184138	13
mTOR signaling pathway	0.007077796	14	0.725976268	4	0.067441344	9	0.077998002	7	0.034069214	13
PPAR signaling pathway	0.000364143	11	0.000197038	10	0.010179646	7	0.008263097	6	1.93212E-05	14
Pyruvate metabolism	0.191688717	3	0.115259819	3	0.032706427	4	0.012291826	4	7.50742E-07	12
p53 signaling pathway	0.090967725	6	0.677731516	2	0.213122638	4	0.099369061	4	0.65280141	3
Phosphatidylinositol signaling system	0.012063792	10	0.113077519	6	0.002263618	10	0.000939587	9	0.000170706	15
Sphingolipid metabolism	0.000108941	10	0.027010105	5	0.08386048	4	0.008219112	5	0.2099859	4
Steroid biosynthesis	0.053126518	3	0.000731424	5	0.028781386	3	0.000181817	5	0.00061686	6
Steroid hormone biosynthesis	0.099921091	4	0.384618981	2	0.730563293	1	0.619992007	1	0.56707676	2
TGF-beta signaling pathway	0.584061319	4	0.824900947	2	0.220264115	5	0.41485102	3	0.988947716	1
VEGF signaling pathway	0.021856082	7	0.870519274	1	0.158462864	4	0.070380945	4	0.350247013	4
Wnt signaling pathway	0.951386512	3	0.239879709	7	0.380932336	6	0.165932671	6	0.03563754	13

### 3.5 DEGs short time-series expression miner (STEM) analysis

**Figure 4:**
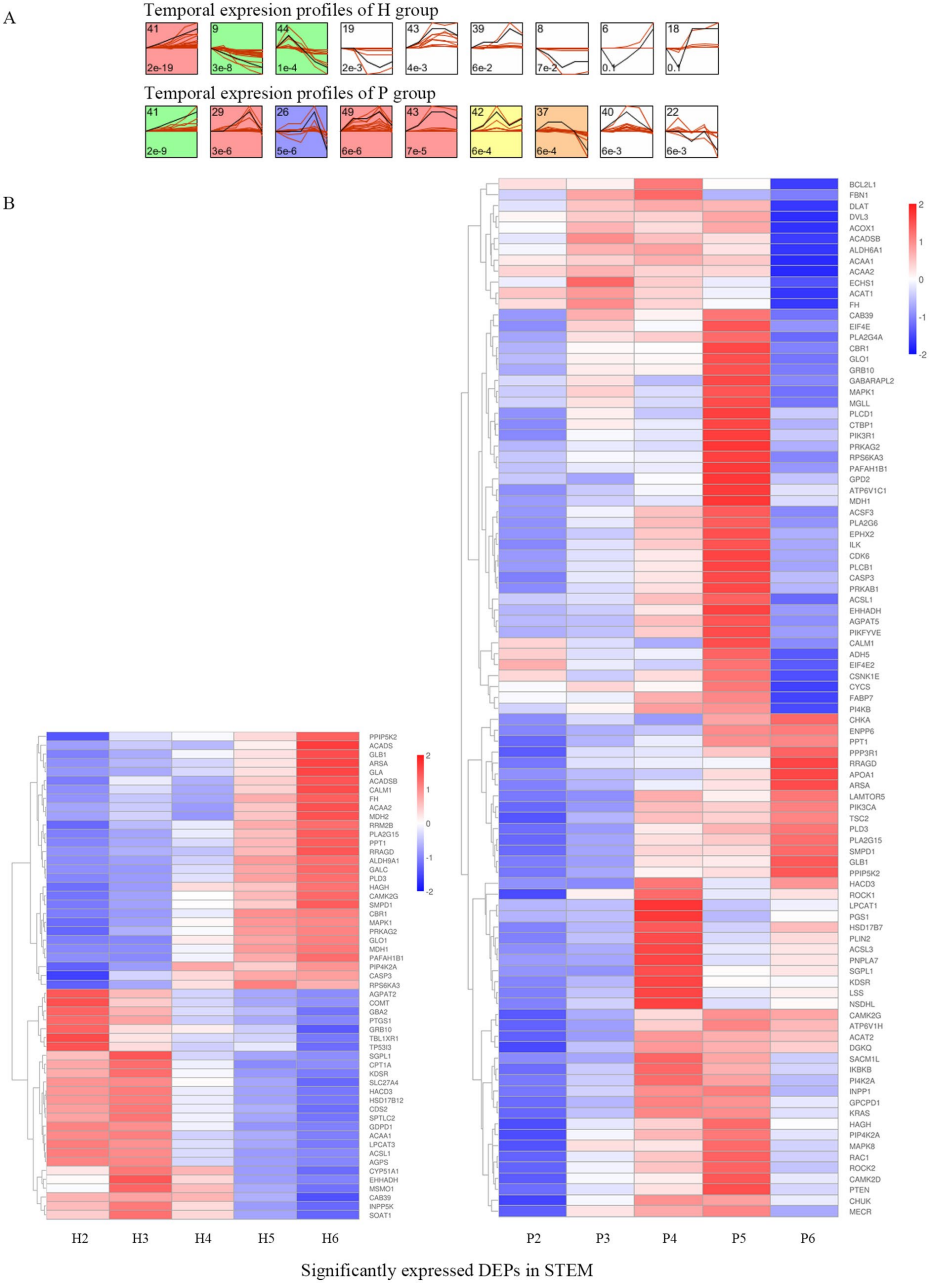
STEM analysis of DEPs. **(A)** The temporal expression profiles of proteins in H and P groups are shown, with the colored backgrounds of the clusters indicating significant changes in the expression trends of proteins within these clusters. A total of 119 proteins exhibited significant expression trends, among which 55 DEPs were identified in the colored clusters of H group, while 95 DEPs were identified in the colored clusters of P group. **(B)** The heatmap of 119 DEPs in the colored clusters is shown, with the left side displaying the heatmap of 55 DEPs from H group and the right side displaying the heatmap of 95 DEPs from P group.

There were 173 DEPs in 23 pathways related to lipid metabolism, and further analysis for STEM analysis of these 173 DEPs showed that a total of 119 DEPs were significantly expressed (Figure 4A). 55 DEPs were significantly expressed in the H group, 95 DEPs were significantly expressed in the P group, and 31 DEPs were significantly expressed in both groups (Table 2). A heat map of the 119 DEPs is shown in Figure 4B. Among them, the expression of Arylsulfatase A (ARSA), Calcium/calmodulin-dependent protein kinase (CAMK2G), Beta-galactosidase (GLB1), Phospholipase A2 group XV (PLA2G15), Phospholipase D family member 3 (PLD3), Inositol hexakisphosphate and Diphosphoinositol-pentakisphosphate kinase (PPIP5K2), Palmitoyl-protein thioesterase 1 (PPT1), Ras-related GTP-binding protein (RRAGD), and Sphingomyelin phosphodiesterase 1 (SMPD1), a total of nine DEPs showed an upward trend in both the H and P groups.

**Table 2:** 119 proteins with significance in STEM.

Group	Profile	Protein description (Gene name)	Number
H	9	Acetyl-CoA acyltransferase 1 (ACAA1), Long-chain-fatty-acid--CoA ligase (ACSL1), 1-acyl-sn-glycerol-3-phosphate acyltransferase (AGPAT2), Alkylglycerone-phosphate synthase (AGPS), Phosphatidate cytidylyltransferase (CDS2), Catechol O-methyltransferase (COMT), Glucosylceramidase beta 2 (GBA2), Glycerophosphodiester phosphodiesterase domain containing 1 (GDPD1), Growth factor receptor bound protein 10 (GRB10), Very-long-chain (3R)-3-hydroxyacyl-CoA dehydratase (HACD3), Hydroxysteroid 17-beta dehydrogenase 12 (HSD17B12), Lysophosphatidylcholine acyltransferase 3 (LPCAT3), Prostaglandin-endoperoxide synthase 1 (PTGS1)M, Solute carrier family 27 member 4 (SLC27A4), Transducin beta like 1 X-linked receptor 1 (TBL1XR1), Tumor protein p53 inducible protein 3 (TP53I3)	16
	41	Acetyl-CoA acyltransferase 2 (ACAA2), Acyl-CoA dehydrogenase short chain (ACADS), Acyl-CoA dehydrogenase short/branched chain (ACADSB), Aldehyde dehydrogenase 9 family member A1 (ALDH9A1), Arylsulfatase A (ARSA), Calmodulin 1 (CALM), calcium/calmodulin-dependent protein kinase (CAMK2G), Caspase 3 (CASP3), carbonyl reductase (NADPH)(CBR1), Fumarate hydratase, mitochondrial (FH), Galactocerebrosidase (GALC), Alpha-galactosidase (GLA), Beta-galactosidase (GLB1), Lactoylglutathione lyase (GLO1), Hydroxyacylglutathione hydrolase (HAGH), mitogen-activated protein kinase (MAPK1), Malate dehydrogenase (MDH1), Malate dehydrogenase (MADH2), Lissencephaly-1 homolog (PAFAH1B1), Phosphatidylinositol-5-phosphate 4-kinase type 2 alpha (PIP4K2A), Phospholipase A2 group XV (PLA2G15), Phospholipase D family member 3 (PLD3), Inositol hexakisphosphate and diphosphoinositol-pentakisphosphate kinase (PPIP5K2), Palmitoyl-protein thioesterase 1 (PPT1), Protein kinase AMP-activated non-catalytic subunit gamma 2 (PRKAG2), Ribosomal protein S6 kinase (RPS6KA3), Ras-related GTP-binding protein (RRAGD), Ribonucleotide reductase regulatory TP53 inducible subunit M2B (RRM2B), Sphingomyelin phosphodiesterase 1 (SMPD1)	29
	44	Calcium binding protein 39 (CAB39), carnitine O-palmitoyltransferase (CPT1A), Cytochrome P450 family 51 subfamily A member 1 (CYP51A1), enoyl-CoA hydratase (EHHADH), Inositol polyphosphate-5-phosphatase K (INPP5K),3-dehydrosphinganine reductase (KDSR), Methylsterol monooxygenase 1 (MSMO1), Sphingosine-1-phosphate lyase 1 (SGPL1), O-acyltransferase (SOAT1), Serine palmitoyltransferase long chain base subunit 2 (SPTLC2)	10
P	26	S-(hydroxymethyl)glutathione dehydrogenase (ADH5), Calmodulin 1 (CALM1), carbonyl reductase (CBR1), Casein kinase 1 epsilon (CSNK1E), Cytochrome c (CYCS), Disheveled segment polarity protein 3 (DVL3), Eukaryotic translation initiation factor 4E family member 2 (EIF4E2), Fatty acid binding protein 7 (FABP7), GABA type A receptor associated protein like 2 (GABARAPL2), Lactoylglutathione lyase (GLO1), Growth factor receptor bound protein 10 (GRB10), mitogen-activated protein kinase (MAPK1), Monoglyceride lipase (MGLL), Phosphatidylinositol 4-kinase beta (PI4KB), Phospholipase A2 (PLA2G4A), Ribosomal protein S6 kinase (RPS6KA3)	16
	29	Acyl-CoA synthetase family member 3 (ACSF3), Long-chain-fatty-acid--CoA ligase (ACSL1), 1-acylglycerol-3-phosphate O-acyltransferase 5 (AGPAT5), V-type proton ATPase subunit C (ATP6V1C1), Caspase 3 (CASP3), Cyclin dependent kinase 6 (CDK6), enoyl-CoA hydratase (EHHADH), Epoxide hydrolase 2 (EPHX2), Glycerol-3-phosphate dehydrogenase (GPD2), Integrin-linked protein kinase (ILK), Malate dehydrogenase (MDH1), Lissencephaly-1 homolog (PAFAH1B1), 1-phosphatidylinositol-3-phosphate 5-kinase (PIKFYVE), phospholipase A2 (PLA2G6), 1-phosphatidylinositol 4, 5-bisphosphate phosphodiesterase (PLCB1), Protein kinase AMP-activated non-catalytic subunit beta 1 (PRKAB1), Protein kinase AMP-activated non-catalytic subunit gamma 2 (PRKAG2)	17
	37	Acetyl-CoA acyltransferase 1 (ACAA1), Acetyl-CoA acyltransferase 2 (ACAA2), Acyl-CoA dehydrogenase short/branched chain (ACADSB), Acetyl-CoA acetyltransferase 1 (ACAT1), Acyl-coenzyme A oxidase (ACOX1), Aldehyde dehydrogenase 6 family member A1 (ALDH6A1), Bcl-2-like protein 1 (BCL2L1), Acetyltransferase component of pyruvate dehydrogenase complex (DLAT), Enoyl-CoA hydratase, short chain 1 (ECHS1), Fibrillin 1 (FBN1), Fumarate hydratase, mitochondrial (FH)	11
	41	Apolipoprotein A1 (APOA1), Arylsulfatase A (ARSA), calcium/calmodulin-dependent protein kinase (CAMK2G), Choline kinase alpha (CHKA), Ectonucleotide pyrophosphatase/phosphodiesterase 6 (ENPP6), Beta-galactosidase (GLB1), Ragulator complex protein LAMTOR5 (LAMTOR5), phosphatidylinositol-4, 5-bisphosphate 3-kinase (PIK3CA), Phospholipase A2 group XV (PLA2G15), Phospholipase D family member 3 (PLD3), Inositol hexakisphosphate and diphosphoinositol-pentakisphosphate kinase (PPIP5K2), Protein phosphatase 3 regulatory subunit B, alpha (PPP3R1), Palmitoyl-protein thioesterase 1 (PPT1), Ras-related GTP-binding protein (RRAGD), Sphingomyelin phosphodiesterase 1 (SMPD1), TSC complex subunit 2 (TSC2)	16
	42	Acyl-CoA synthetase long chain family member 3 (ACSL3), Very-long-chain (3R)-3-hydroxyacyl-CoA dehydratase (HACD3), Hydroxysteroid 17-beta dehydrogenase 7 (HSD17B7), 3-dehydrosphinganine reductase (KDSR), Lysophosphatidylcholine acyltransferase 1 (LPCAT1), Terpene cyclase/mutase family member (LSS), NAD (P) dependent steroid dehydrogenase-like (NSDHL), CDP-diacylglycerol--glycerol-3-phosphate 3-phosphatidyltransferase (PGS1), Perilipin (PLIN2), Patatin like phospholipase domain containing 7 (PNPLA7), non-specific serine/threonine protein kinase (ROCK1), Sphingosine-1-phosphate lyase 1 (SGPL1)	12
	43	Acetyl-CoA acetyltransferase 2 (ACAT2), V-type proton ATPase subunit H (ATP6V1H), Inhibitor of nuclear factor kappa-B kinase subunit alpha (CHUK), Diacylglycerol kinase (DGKQ), Glycerophosphocholine phosphodiesterase 1 (GPCPD1), IkappaB kinase (IKBKB), Inositol polyphosphate-1-phosphatase (INPP1), KRAS proto-oncogene, GTPase (KRAS), Phosphatidylinositol 4-kinase type 2 (PI4K2A), SAC1 like phosphatidylinositide phosphatase (SACM1L)	10
	49	Calcium binding protein 39 (CAB39), calcium/calmodulin-dependent protein kinase (CAMK2D), C-terminal binding protein 1 (CTBP1), Eukaryotic translation initiation factor 4E (EIF4E), Hydroxyacylglutathione hydrolase (HAGH), Stress-activated protein kinase JNK (MAPK8), Mitochondrial trans-2-enoyl-CoA reductase (MECR), Phosphatidylinositol 3-kinase regulatory subunit alpha (PIK3R1), Phosphatidylinositol-5-phosphate 4-kinase type 2 alpha (PIP4K2A), Phosphoinositide phospholipase C (PLCD1), Phosphatidylinositol 3,4,5-trisphosphate 3-phosphatase and dual-specificity protein phosphatase PTEN (PTEN), Rac family small GTPase 1 (RAC1), Rho-associated protein kinase (ROCK2)	13

### 3.6 Protein–protein interaction (PPI) network analysis

**Figure 5:**
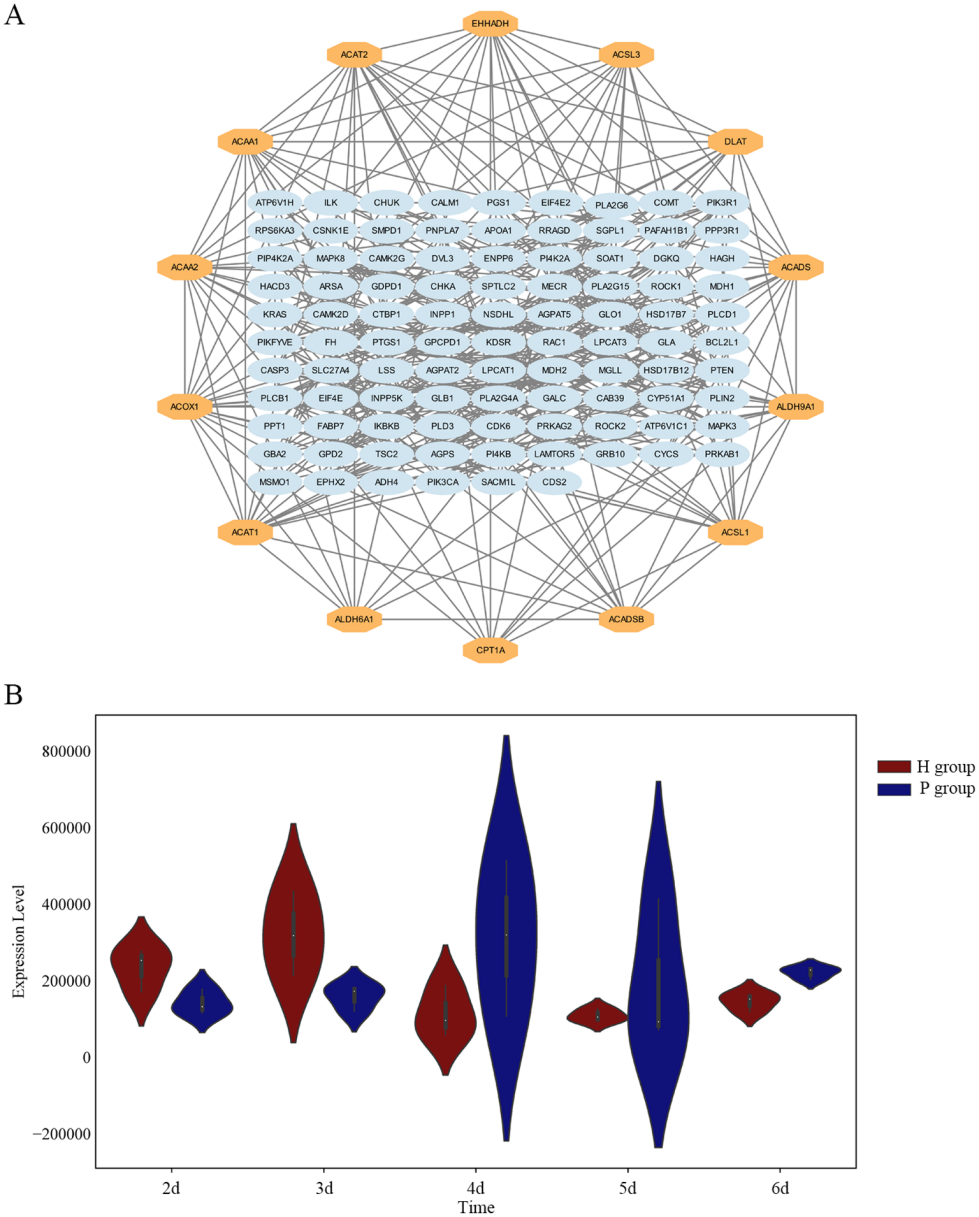
Screening of key proteins. **(A)** Protein–protein interaction (PPI) network analysis for DEPs, the 14 proteins in the outer circle are the hub proteins. **(B)** The violin plot illustrates the distribution of ACSL3 protein expression levels. Across the five time points, ACSL3 protein expression levels show significant differences between H and P groups.

A protein–protein interaction (PPI) network was constructed using the network visualization resource STRING for 119 DEPs, as shown in Figure 5A. Fourteen key proteins were screened, including Long-chain acyl-CoA synthetases family members (ACSL1, ACSL3), Acetyl-CoA acetyltransferase (ACAT1, ACAT2), Acetyl-CoA acyltransferase (ACAA1, ACAA2), Carnitine O-acyltransferase CPT1 (CPT1A), Acyl-CoA oxidase (ACOX1), Short-chain acyl-CoA dehydrogenase (ACADS, ACADSB), Aldehyde dehydrogenase family members (ALDH6A1, ALDH9A1), Enoyl-CoA hydratase (EHHADH), and Dihydrolipoamide acetyltransferase (DLAT). 12 of these proteins were involved in the fatty acid degradation process, suggesting that fatty acid degradation plays a key role in lipid metabolism in hierarchical follicles and pre-hierarchical follicles. ACSL3 is a key rate-limiting protein in fatty acid degradation and is a repeatedly identified LD protein. In this study, ACSL3 was differentially expressed in both phases (Figure 5B), and there was a highly significant difference in ACSL3 expression at 6 d of cell culture.

## 4 Discussion

The development of avian follicles follows a strict hierarchy. During the progression from pre-hierarchical follicles to hierarchical follicles, there is a gradual deposition of yolk precursors (primarily lipids) within the follicles. Cellular neutral lipids are stored in the form of LDs, which may play a more significant role in the development of goose follicles. LDs have been reported to be associated with follicular development, with the size and number of LDs changing as the follicle grows, total intracellular lipid content also increases gradually with follicular growth (35–37). In geese, differences in the morphology and lipid content of LDs have been reported in hierarchical and pre-hierarchical granulosa cells (13). In the present study, we found highly significant differences in LDs in granulosa cells of goose follicles at different stages of development. The content of LDs was significantly higher in hierarchical granulosa cells than in pre-hierarchical granulosa cells, and hierarchical granulosa cells tended to synthesize large LDs, whereas pre-hierarchical granulosa cells tended to form smaller LDs. The content of LDs in hierarchical granulosa cells gradually increased with increasing culture time, reaching a maximum after 5 d of cell culture and then decreasing. These findings are consistent with previous reports.

The functions of LDs are primarily carried out by LDAPs, and previous studies have identified hundreds of these proteins within LDs. In this study, we found a large number of enzymes involved in lipid synthesis processes, such as FASN, ACSFs, ACSLs, DGKs, and MSMO1, these enzymes are found in both hierarchical granulosa cells and pre-hierarchical granulosa cells. This also suggests an important role for LD lipid metabolism in granulosa cells. FASN is a key enzyme in fatty acid synthesis in mammals, with mRNA and protein expression observed in rat, bovine, and human granulosa cells (38). Previous studies have identified FASN in the granulosa layer of the goose small white follicle and small yellow follicle by using transcriptome sequencing (39). ACSF, an isoenzyme of ACS, has been shown to be involved in fatty acid and cholesterol synthesis (40) and is associated with egg production in poultry (41, 42). Long-chain acyl-coenzyme A synthases (ACSLs) are key enzymes in the regulation of lipid metabolism, including fatty acid elongation, oxidative decomposition, and phospholipid generation (43). ACSL3 localizes to LDs and promotes their biogenesis (44). We also previously identified ACSL3 mRNA expression in the granulosa cells of goose hierarchical follicles (45). Diacylglycerol kinase (DGK) is a lipid kinase converting diacylglycerol (DAG) to phosphatidic acid (PA) and can be involved in various cellular responses by regulating balance of the two lipid messengers (46). Expression of DGK protein has been found in both the ovary and uterus of mice, suggesting a role for DGK in the maturation of mouse oocytes (47). MSMO1 gene encodes an enzyme involved in cholesterol biosynthesis. Cholesterol and intermediates in its biosynthetic pathway are required for many developmental processes including membrane biosynthesis, steroid biosynthesis, and adipogenesis (48). DEPs mentioned above were involved in the fatty acid synthesis process, confirming that both goose hierarchical follicles and pre-hierarchical follicles have fatty acid synthesis function (14), and that there are some differences in the lipid synthesis process between hierarchical follicles and pre-hierarchical follicles. These DEPs may influence goose follicle development by regulating lipid synthesis in LDs, which in turn modulates cellular lipid metabolism.

In addition to enzymes involved in lipid metabolism, LDs are also enriched with various other proteins that participate in multiple cellular life activities. This study identified differential proteins associated with steroid hormone secretion and cell proliferation and apoptosis in both hierarchical and pre-hierarchical granulosa cells. These differential proteins may influence follicular development by regulating steroid hormone secretion, cell proliferation, and apoptosis.

Avian follicles exhibit a unique hierarchical development, where small pre-hierarchical follicles are unable to synthesize active steroid hormones using steroids as substrate, and steroid hormone secretion occurs only in the hierarchical granulosa cells. Among the top 20 up-regulated DEPs in this study, NPC2 is an intracellular cholesterol transporter protein, which has the intracellular activity of extracting cholesterol from low-density lipoproteins (LDL) and delivering it to membrane-bound NPC1, which cooperates with downstream proteins to transport cholesterol throughout the cell to fulfill its biological function (49). Previous research on chicken small yellow and large yellow follicles revealed a six-fold upregulation of NPC2 mRNA expression (50). Similarly, our study found significant upregulation of NPC2 protein at all five time points in both H and P groups. MGLL, a member of the serine hydrolase superfamily, is localized to LDs and plays a key role in breaking down monoacylglycerol into free fatty acids and glycerol (47). It also hydrolyzes 2-arachidonoylglycerol (2-AG) to arachidonic acid (AA). Additionally, RDH10 is also localized in LDs and binds to their surface during acyl ester biosynthesis (49). We hypothesized that lipids in the LDs of hierarchical follicles are degraded by hydrolytic enzymes, such as MGLL. The degradation product, cholesterol, is then transported by transporter enzymes, which are involved in the process of steroid hormone secretion. This hypothesis confirms the differences in steroid hormone secretion between avian hierarchical follicles and pre-hierarchical follicles. Granulosa cells promote cholesterol biosynthesis and hydrolysis through different enzymes, ensuring a steady supply of cholesterol for steroidogenesis, reflecting their ability to respond to hormonal signals and provide energy for follicular development. Among the down-regulated top 20 DEPs, we identified several proteins related to the cytoskeleton and structure, such as NEFL, FLNC, LSP1, PGM5. The movement of LDs is primarily driven by associated proteins such as the cytoskeleton, suggesting that in hierarchical follicles, the reduced expression of these proteins may lead to decreased movement of LDs and reduced contact with other organelles. Our findings align with the observed differences in lipid metabolism and steroid hormone secretion between hierarchical and pre-hierarchical follicles.

With increasing incubation time, we found that the expression of certain proteins showed a continuous upward trend, among which CAMK2G, RRAGD, SMPD1 and PPIP5K2 were closely related to cell proliferation and apoptosis, potentially playing essential roles in the development of goose follicles. Intracellular calcium ions regulate steroidogenesis in both the testis and the ovary, whereas CAMK2G, which is involved in the calcium signaling pathway, regulates apoptosis and also acts as a regulator of cell adhesion, proliferation, and survival (51). Its expression has been detected in the ovaries of mice, goats, and pigs (52, 53). CAMK2G is known to control oocyte activation by restoring the cell cycle in mouse oocytes (54, 55). RRAGD mediates amino acid stimulation of the mTOR signaling pathway, a crucial pathway for cell growth and proliferation (56). SMPD1 protects human luteinizing granulosa cells from apoptosis and plays a vital role in initiating female germ cell death signaling (57). PPIP5K2 is highly expressed in colorectal cancer (CRC) and enhances the proliferation and migration abilities of CRC cells (58). These DEPs showed a progressive increase in protein expression with time in H and P groups and were significant findings in STEM analysis, which may be important for follicular development. We propose that these DEPs may play similar roles in avian species, further affecting goose follicle development by controlling a range of life activities such as granulosa cells proliferation and apoptosis.

Finally, we performed PPI network analysis and identified 14 candidate key proteins that may regulate lipid metabolism in granulosa cells. After pathway analysis, we found that 12 of these proteins were involved in the fatty acid degradation process, and therefore hypothesized that the fatty acid degradation pathway might be an important contributor to the differences in LDs of granulosa cells at different developmental stages. Fatty acid oxidation (FAO) is the main pathway for fatty acid degradation, and CPT1A is the key rate-limiting enzyme in FAO. It has been reported that knockdown of CPT1A induces lipid differentiation in chicken adipocytes, inhibits the *β*-oxidation process, and promotes the formation of LDs (59). The ACAA gene encodes an enzyme of the thiolase family, and in sheep preadipocytes, overexpression of ACAA1 reduced cellular lipid accumulation and triglyceride content (60), while lack of ACAA2 expression may also lead to reduced LD formation (61). ACAA1 and ACAA2 can be involved in fatty acid elongation and degradation by catalyzing the final step of the β-oxidation pathway and are therefore essential for energy production and lipid metabolism (62). In addition, ACOX1, EHHADH, and ACAD are enzymes involved in the fatty acid β-oxidation pathway. One of the main functions of LDs is to produce energy through the fatty acid β-oxidation pathway by oxidizing free fatty acids. Mitosis is prevalent in pre-hierarchical granulosa cells and requires large amounts of energy to supply the metabolism of these cells. It can be speculated that in pre-hierarchical granulosa cells, fatty acids are oxidized and used primarily to generate energy for autotrophic requirements rather than synthesizing LDs.

ACSLs are a group of rate-limiting enzymes in fatty acid metabolism. They are essential for the activation of long chain fatty acids (LCFA). Once esterified by ACSL, LCFAs can enter various metabolic pathways, including cellular fatty acid catabolism, phospholipid synthesis, TG synthesis and CE synthesis. The mammalian ACSLs family contains five members, including ACSL1, ACSL3, ACSL4, ACSL5, and ACSL6. ACSL1 promotes fatty acids uptake and free fatty acids deposition, also affecting TG levels through the PPARγ pathway (63). ACSL3, frequently identified as an abundant LDAP (64). Intracellularly, ACSL3 is predominantly distributed in the periphery of LDs and on the cytoplasmic surface of the endoplasmic reticulum (44). ACSL3 is a key enzyme for LD biogenesis that redistributes from the endoplasmic reticulum to LDs during LD formation, and this localization mechanism of ACSL3 is associated with Rab18 and PLIN2. Rab18 interacts with PLIN2 and forms a complex with ACSL3, promotes the localization of ACSL3 on the surface of nascent LDs, thereby increasing the content of intracellular LDs (65). Addition of oleic acid (OA) to human hepatocyte medium enhances intracellular LD accumulation, accompanied by increased ACSL3 expression, while ACSL inhibitors Triacsin C reduce LD content, indicating that ACSL is involved in neutral lipid synthesis and LD formation (66). Recently, it was found that ACSL3 not only regulates the biogenesis of LDs in cells, but also decreases the susceptibility of cells to ferroptosis (67). In conclusion, ACSL3 is involved in intracellular lipid metabolism by promoting the formation and maturation of LDs. Expression of ACSL3mRNA is currently detected in granulosa cells from humans (1), bovine (68), goose (45). Recent evidence suggests that ACSL3 is important in maintaining the channeling of extracellularly-derived lipids to FAO (69). In our study, ACSL3 protein was expressed in both hierarchical follicles and pre-hierarchical follicles, and protein expression changed with time. In pre-hierarchical follicles, lipid metabolism in granulosa cells primarily focuses on maintaining cellular structure and basal metabolism. During this phase, granulosa cells exhibit weak lipid synthesis capacity, limited lipid accumulation, and low activity of lipid metabolism-related enzymes. The main functions of lipids are to construct cell membranes and provide energy. In contrast, lipid metabolism in granulosa cells of hierarchical follicles is significantly enhanced, particularly in the synthesis and accumulation of yolk precursor substances, to meet the demands of yolk formation and rapid follicular development. During this phase, the activity of lipid synthesis-related enzymes is markedly increased, and lipids accumulate extensively in the form of TG and CE. Therefore, we hypothesize that in goose hierarchical follicular granulosa cells, most of the fatty acids activated by ACSL3 are utilized for the synthesis of TG and CE, which further contribute to the formation of LDs, ultimately leading to differences in lipid deposition.

## 5 Conclusion

**Figure 6:**
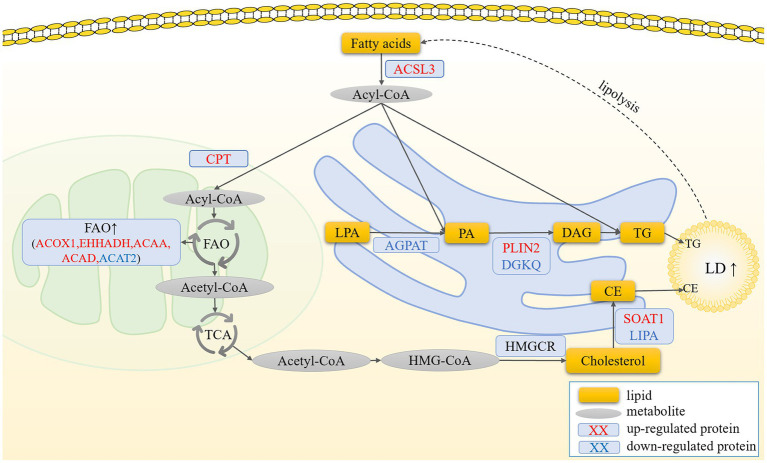
Prediction of the molecular mechanism by which ACSL3 regulates the synthesis of LDs in granulosa cells. In pre-hierarchical granulosa cells, fatty acids activated by ACSL3 are oxidized and enter the TCA cycle to meet the cells’ energy demands. In contrast, in hierarchical granulosa cells, fatty acids activated by ACSL3 promote lipid droplet formation through the synthesis of TG and CE, leading to a significant increase in intracellular lipid deposition.

In conclusion, we observed significantly higher levels of LDs in hierarchical granulosa cells than in pre-hierarchical granulosa cells. Moreover, the content of LDs in granulosa cells at different developmental stages underwent dynamic changes with the extension of cell culture time. Importantly, this study marks the first proteomic analysis of LDs in goose follicle granulosa cells, offering detailed insights into the primary components of LDAPs. These foundational data serve as a valuable reference for future investigations into LDs in geese. Furthermore, we identified ACSL3, a crucial regulator of lipid metabolism in granulosa cells, and hypothesize that ACSL3 acts as a pivotal modulator of lipid variations in granulosa cells across different developmental stages. Specifically, in pre-hierarchical granulosa cells, fatty acids activated by ACSL3 are primarily oxidized and enter the TCA cycle to meet the energy demands of the cells. In contrast, in hierarchical granulosa cells, fatty acids activated by ACSL3 are utilized for the synthesis of TG and CE, further promoting the formation of LDs. This process leads to a significant increase in lipid deposition within hierarchical granulosa cells (Figure 6). However, the precise regulatory mechanisms underlying these processes remain to be elucidated (70).

## Data Availability

The mass spectrometry proteomics data have been deposited in the ProteomeXchange Consortium through the PRIDE partner repository with the dataset identifier PXD061272.

## Footnotes

^1^ https://www.ncbi.nlm.nih.gov/research/cog

^2^ https://www.geneontology.org

^3^ https://www.genome.jp/kegg

^4^ https://string-db.org/

## Supplementary material

SUPPLEMENTARY FIGURE S1 The top 20 most significantly enriched GO terms and KEGG pathways.

SUPPLEMENTARY TABLE S1 Functional classification of DEPs.

SUPPLEMENTARY TABLE S2 The top 20 up and down-regulated proteins at five time points.

SUPPLEMENTARY TABLE S3 The GO terms and KEGG pathways at five time points.
