# Supplementary figures and images for: Proteome of granulosa cells lipid droplets reveals mechanisms regulating lipid metabolism at hierarchical and pre-hierarchical follicle in goose
Source: Front Vet Sci. 2025 Mar 31;12:1544718. doi: 10.3389/fvets.2025.1544718 (PMC11995638; doi:10.3389/fvets.2025.1544718)

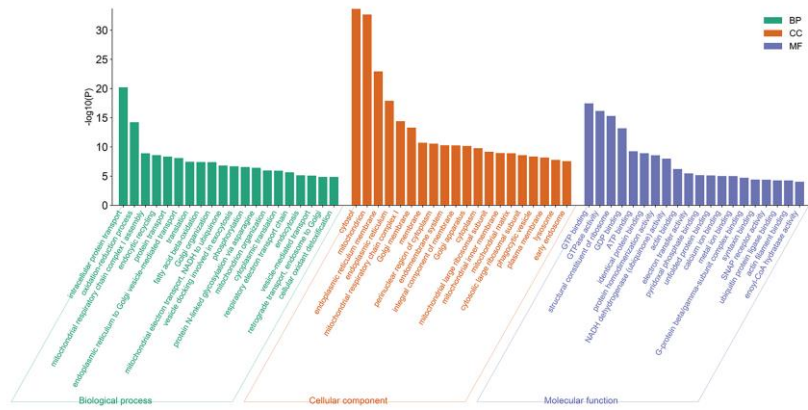

H2 vs P2

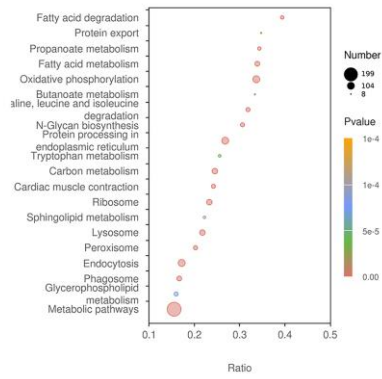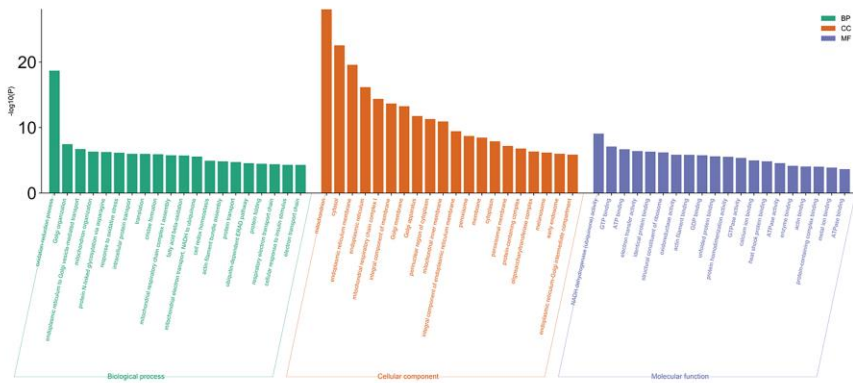

H3 vs P3

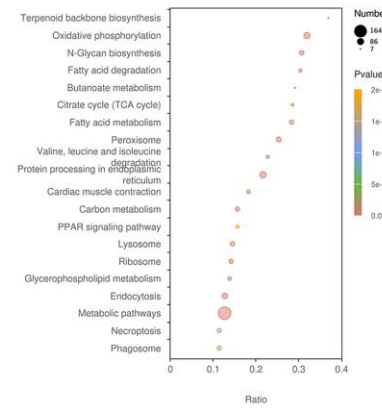

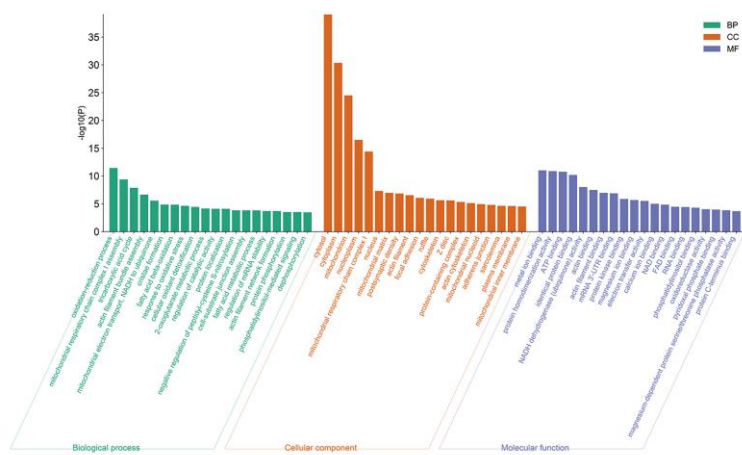

H4 vs P4

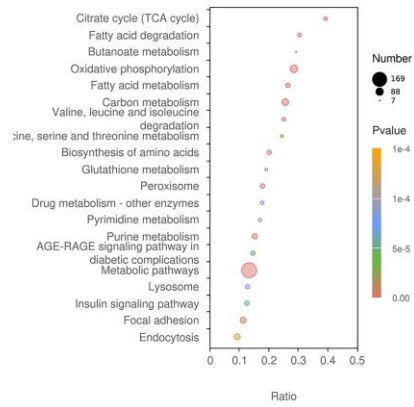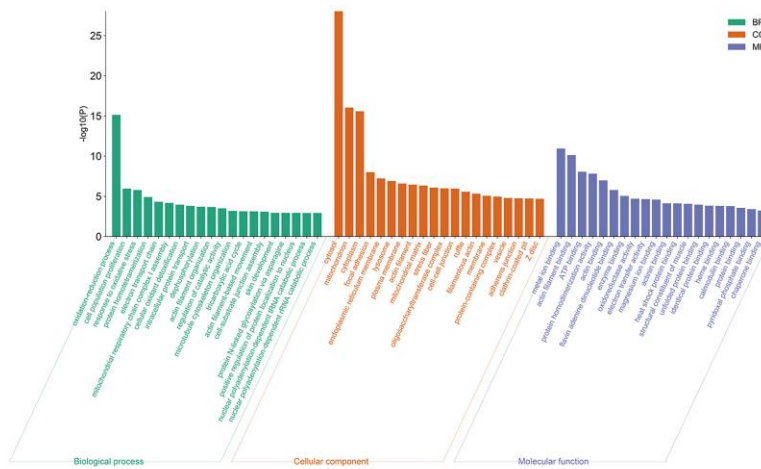

H5 vs P5

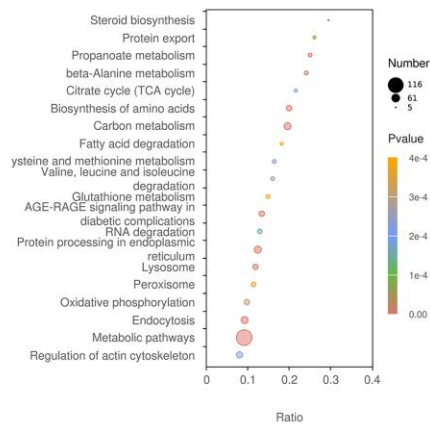

Supplement: SUPPLEMENTARY FIGURE S1 — The top 20 most significantly enriched GO terms and KEGG pathways. [file Data_Sheet_1.zip › Additional file/Figure.S1.pdf]
